# Assessment of Mental Health Nurses’ Competences: A Scoping Review

**DOI:** 10.62641/aep.v54i3.2069

**Published:** 2026-06-15

**Authors:** Javier Sanz Calvo, Mariana Alina Renghea, Mercedes Gómez del Pulgar García-Madrid

**Affiliations:** ^1^Mental Health Center Hortaleza, Psychiatry and Mental Health Department, Ramón y Cajal University Hospital, 28033 Madrid, Spain; ^2^Faculty of Health Sciences, Francisco Vitoria University, Pozuelo de Alarcón (Madrid), 28223 Madrid, Spain

**Keywords:** clinical competence, psychosocial nursing, mental health nursing, competencybased education, nursing evaluation research

## Abstract

**Background:**

Research has explored how undergraduate nursing students or Registered Nurses acquire and assess competences through simulations or training programs. However, there is limited evidence regarding Mental Health Nurses (MHNs) in regular clinical practice. This review aims to identify the clinical competences—knowledge, skills, or attitudes—of MHNs in their clinical practice, and how these are evaluated.

**Methods:**

A scoping review was conducted across four databases (PubMed, CINAHL, MEDLINE, PsycInfo) adhering to the Preferred Reporting Items for Systematic Reviews and Meta-Analyses Extension for Scoping Reviews (PRISMA-ScR) extension guidelines, applying a double screening process.

**Results:**

Twenty-three studies were selected, focusing on nursing competency acquisition programs and assessments tools. The quality of the included studies was deemed acceptable. The findings indicated that educational programs improved professional competences. Among the assessment tools identified, sixty-six were validated while thirty-four were researcher-developed. Most tools targeted core MHN competences, such as physical health (Physical Health Attitude Scale for Mental Health Nurses) and psychiatric emergencies (Attitudes towards Containment Measures Questionnaire). Fewer tools addressed crosscutting competences (Clinical Competency of Mental Health Nursing).

**Conclusions:**

There is a clear need to develop tools specifically designed to assess mental health nursing within Specialized Health Training Programs. To strengthen competency acquisition and evaluation, it is recommended that complementary methods, such as simulations, feedback, and targeted tools, be integrated into MHN training and assessment.

## Introduction

Professional competences can be defined as “the dynamic integration of knowledge, skills, attitudes and values, mobilized effectively to address real-world challenges and deliver optimal solutions within available resources” [1, 2]. This definition can be applied across many disciplines, including nursing, where competence acquisition and assessment are critical.

Research on competence acquisition among undergraduate nursing students has explored diverse techniques and methods, such as supervised clinical placements, simulations, role-play, short training courses and self-directed learning. Similarly, a range of assessment tools has been developed to evaluate nursing competences across different settings [1, 3, 4].

Within the field of mental health, nursing, studies frequently examine the competences of Mental Health or Psychiatric Nurses [5, 6, 7, 8], often focusing on professionals in psychiatric hospitals, regardless of whether they have specialized healthcare qualifications [9].

However, rigorous and objective assessments of professional competences among nurses specialized in Psychiatry or Mental Health, particularly in routine professional practice or through structured training programs, remain limited [7].

Official frameworks, such as the Mental Health Nursing Training Program (Order SPI/1356/2011), outline evaluation criteria and activities but fail to specify assessment methods and protocols. Current evaluations are carried out according to the Resolution of March 21, 2018, establishing basic guidelines for evaluating medical and nursing residents. However, this official framework is criticized for its broad, one-size-fits-all approach, which may not adequately address the specific nuances of mental health nursing or ensure objectivity [10].

As a result, robust evidence on evaluating nursing competences, whether acquired during training or through routine clinical practice, among mental health nurses is scarce. Existing studies primarily target students or registered nurses working in mental health, often relying on simulations rather than real clinical practice [11].

This review aims to identify, compare and synthesize peer-reviewed published evidence on the clinical competences (knowledge, attitude or skill) of mental health nurses in clinical practice, and the methods used to evaluate them.

## Methods

This scoping review aimed to map the competences of Mental Health Nurses (MHNs) as recognized by international professional bodies and to compile all existing evaluation tools documented in literature on professional competences. The review was guided by the central research question: How are the clinical competences of Mental Health Nurses evaluated?

Scoping reviews are particularly suited to exploratory objectives such as these [12] as they allow for a broad examination of diverse literature, although this breadth can hinder a systematic critical appraisal of the evidence [13, 14]. Moreover, scoping reviews are especially valuable in exploring complex fields such as mental health nursing in clinical practice [12, 14].

Although scoping reviews are inherently exploratory, a number of studies were excluded from the sample due to the large volume of literature available and the poor methodological quality of much research on mental health nursing competences.

Manuscripts that had not undergone formal peer review were also omitted. This approach ensured that only rigorous, methodologically sound and peer-reviewed sources informed our mapping.

### Design

This study employed a scoping review to examine the scientific literature on evaluating competences in mental health nursing, specifically those acquired through clinical practice by MHNs or Psychiatric Nurses. The review adhered to the updated guidance by Pollock *et al*. [14], incorporating the Joanna Briggs Institute scoping review (JBI ScR) framework and the Preferred Reporting Items for Systematic Reviews and Meta-Analyses Extension for Scoping Reviews (PRISMA-ScR) extension [12] for systematic scoping reviews. The design also followed the recommended “PCC” framework (Population, Concept, Context) to structure the inquiry [15].

- Population: Mental Health Nurses or Psychiatric Nurses.

- Concept: Methods used to assess the professional or clinical competences (knowledge, skills, or attitudes) of MHNs.

- Context: All clinical practice settings involving Mental Health Nurses.

Finally, the research questions were designed following the PICO (Population, Intervention, Comparison, Outcome) framework (Table 1):

**Table 1.** **Complete search strategy in database according to the PICO framework**.

Population	Intervention	Comparison	Outcomes
“mental health nurs*” OR “psychiatric nurs*”	“competenc* assess*” OR assess* OR “competencies based education” OR “continuing education”	NOT simulat*	“clinical competenc*” OR “competenc*” OR knowledge OR skills

*: a search strategy to include all word endings. PICO, Population, Intervention, Comparison, Outcome.

- Population: Mental Health Nurses or Psychiatric Nurses.

- Intervention: Assessment of competence in clinical practice, through competency-based education or continuing education programs.

- Comparison: none.

- Outcome: clinical competences (knowledge, skills, or attitudes) and competence assessment tools used in evaluation.

### Population and Scope

This review focused specifically on MHNs whose training, skills, and evaluations are distinct and more specialized compared to generalist nurses, reflecting the unique demands of mental healthcare. To ensure a comprehensive yet focused analysis, the search was limited to studies published over the past two decades. This timeframe was selected due to the anticipated scarcity of relevant research and the absence of any previous synthesis of peer-reviewed data on their assessment of MHNs.

### Search Strategy and Inclusion Criteria

The search protocol is detailed in Table 2. Initial searches were conducted in March 2024 using combinations of keywords (see Table 2) and MeSH terms to ensure a comprehensive exploration of the databases. Boolean operators AND (to combine concepts), OR (to include any of the specified terms), and NOT (to exclude irrelevant keywords) were applied to refine the results. A second main search was performed in April 2024, with the final database search strategy aligned with the PICO framework (Population, Intervention, Comparison, Outcomes), as shown in Table 1.

**Table 2.** **Inclusion criteria**.

Domain	Inclusion criteria	Exclusion criteria
Time period	2012–2023	
Setting	All clinical mental healthcare settings, such as primary care, hospital or residence care	Non-clinical mental healthcare settings: university and academic settings
Type of study design	All peer-reviewed studies such as quantitative studies, qualitative studies or mixed methods. Open access	Non-peer-reviewed studies and editorials, letters to the editor, descriptive studies, opinion studies, literature reviews, commentaries and any papers not reporting data
Population	Registered Mental Health Nurses and Psychiatric Nurses. Registered Nurses working in Psychiatry	Students, Nurses, Nurse Practitioners, Emergency Nurses, Physicians, Doctors, Psychologist, Social workers and other non-mental health professionals. * In the case of finding studies with heterogeneous populations, they will only be included when most of the sample are mental health nurses
Reported outcomes	Assessment of competences of Mental Health Nursing in clinical practice. Mental Health Nurses being assessed for knowledge, skills or attitudes in clinical practice. Competence Assessment Tools for Mental Health Nurses. Assessment Training Programs for Mental Health Nurses	Non-clinical practice-based. Do not assess clinical competences. Mental Health Nurses clinical roles or professional identity Online competences*. E-learnings programs*. Gain simulation competences or skills
Language	English, Spanish	Other languages
Databases	PubMed, CINAHL complete, MEDLINE and PsycInfo	Other databases
Keywords (MeSH)	Clinical Competence, Psychosocial Nursing, Mental Health Nursing, Competency Based Education, Nursing Evaluation Research	

* Online competences or e-learning programs were excluded from this review. These approaches often prioritize knowledge acquisition over the development of practical skills and competences, and present challenges in assessment, and are not sufficiently integrated into real-world clinical practice.

To minimize omissions, an open manual search was also conducted in university repositories and academic search engines, with minor adjustments to search terms to capture any additional key studies.

### Study Selection

In this scoping review, the relevance of each search result was evaluated through peer review. After removing duplicate records, two independent reviewers screened titles and abstracts against predefined inclusion and exclusion criteria, excluding any studies unrelated to mental health nursing competences. Discrepancies were resolved through discussion, with a third reviewer consulted if consensus could not be reached.

Potentially relevant studies then underwent full-text assessment by the same reviewers, following the same protocol for resolving disagreements.

The entire selection process was documented in a PRISMA-ScR flow diagram, detailing the number of studies identified, screened, excluded, and included. In line with JBI ScR guidelines for scoping reviews, which prioritize mapping evidence over quantifying inter-rater reliability, Cohen’s kappa coefficient was not calculated. Instead, reliability was ensured through independent dual screening and extraction, with discrepancies resolved by consensus and, when necessary, verified by a third reviewer.

### Data Extraction and Synthesis

Data extraction was performed using a custom-designed template, which was piloted by a second reviewer. Two reviewers independently extracted the following details from the selected studies: bibliographic characteristics (author, year and country), methodology (aim, study design and sample), main results, and main outcomes (type of competences, topic of competences and measurement instrument).

## Results

### Search Results

**Fig. 1.**
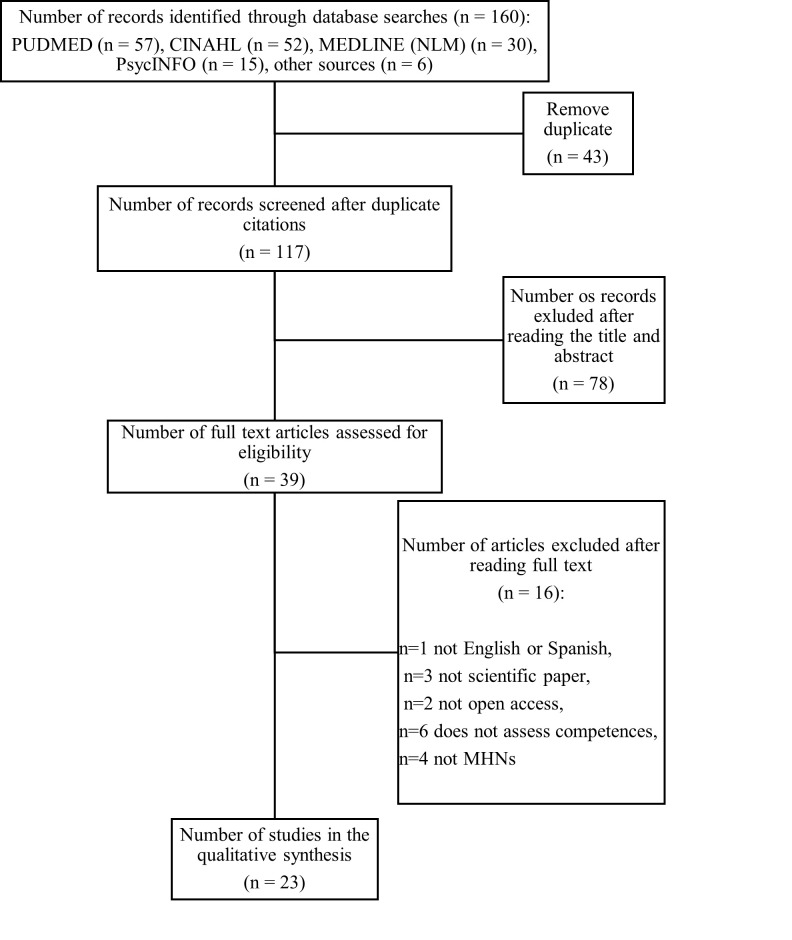
**PRISMA-ScR Preferred Reporting Items for Systematic Reviews and Meta-Analyses Extension for Scoping Reviews guidelines flow diagram**.

Database searches initially identified 160 records, of which 43 were duplicates. After applying selection criteria and screening by title and abstract, 78 studies were excluded, leaving 39 studies for full-text review. Of these, 16 were excluded for various reasons, such as not assessing professional competences (Fig. 1). Ultimately, 23 scientific studies were included in the analysis.

### Study Characteristics and Methods

A total of twenty-three studies were included in this review: six were systematic reviews including qualitative or mixed-method studies, with limited internal homogeneity (n = 6). The remaining 17 were primary studies, none of which were randomized (n = 6 quasi-experimental, n = 7 cross-sectional, and n = 4 validation studies). While the methodology of primary studies was considered acceptable, the authors consistently noted the poor quality of the included studies and stressed the need for more rigorous research.

The studies were published in English (n = 22) and Spanish (n = 1), published between 2013 and 2023. They were conducted across 13 countries, including UK (n = 6), Japan (n = 3), Iran (n = 2), Korea (n = 2), Australia (n = 2), Saudi Arabia (n = 1), Canada (n = 1), Portugal (n = 1), Palestine (n = 1), Turkey (n = 1), Taiwan (n = 1), USA (n = 1), and Spain (n = 1) (Table 3, Ref. [9, 16, 17, 18, 19, 20, 21, 22, 23, 24, 25, 26, 27, 28, 29, 30, 31, 32, 33, 34, 35, 36, 37]).

**Table 3.** **Summary of included studies on mental health nursing competences**.

Author, year, country	Aim	Study design	Sample	Findings
Robson and Haddad (2012) [16] United Kingdom	To describe attitudes, confidence, clinical practice, and perceived training needs among qualified mental health nurses regarding the physical healthcare of people with severe and enduring mental illness	Cross-sectional study	585 MHNs^1^	Staff frequently provided advice on diet and exercise but less often on cancer screening and smoking cessation. Nurses with additional training or qualifications showed more positive attitudes towards physical healthcare. Overall, attitudes were positive
Hemingway *et al*. (2013) [17] United Kingdom	To increase knowledge and skills of MHNs in managing diabetes	Quasi- experimental	9 MHNs^1^	Workshops improved knowledge and skills for assessing and managing diabetes, improving the physical healthcare for people with a serious mental illness
Park and Jung (2014) [18] Korea	To develop and validate an expert competency model scale for preventing addictive adolescent behavior and identify educational needs of psychiatric mental health nurses	Reliability and validity study	137 PNs^2^	The expert competency model for preventing adolescent addictive behavior included: - Positive self-worth - Adolescent comprehension of their environment - Capability of inner motivation - Perception of personal competency - Communication skills with adolescents - Addiction comprehension capability - Addiction management skills The scale demonstrated good reliability and validity. The highest educational needs were addiction management and adolescent communication skills
White *et al*. (2014) [19] United Kingdom	To develop and evaluate a brief evidence-based education program to enhance physical health knowledge and skills in mental health nurses	Quasi- experimental	38 MHNs^1^	Statistically significant knowledge gain post-workshop, participants expressed satisfaction and willingness to apply learning to their practice
Hemingway *et al*. (2015) [20] United Kingdom	To increase understanding and skills of mental health nurses working with diabetes persons	Quasi- experimental	26 MHNs^1^	Workshops motivated nurses to improve their health literacy in diabetes care
Matsuda and Kono (2015) [21] Japan	To develop and evaluate a psychoeducational training program for nursing competences	Quasi- experimental	38 MHNs^1^	Knowledge and attitudes of MHNs improved but no significant change in psychoeducational skills, self- efficacy or motivation
de Almeida Vieira Monteiro *et al*. (2016) [22] Portugal	To test the psychometric properties of the Portuguese version of the Multicultural Mental Health Awareness Scale— (MMHAS)	Reliability and validity study	306 MHNs^1^	The MMHAS Portuguese version is effective for evaluating multicultural competency training programs
Dickens *et al*. (2016) [23] United Kingdom	To explore interventions improving mental health nursing competences towards borderline personality disorder	Systematic Review with 9 studies	>1.100 PNs^2^ or nurses working in psychiatry	Greatest improvements in knowledge and attitudes of MHNs towards borderline personality disorder, especially among those without previous training
AL-Sagarat *et al*. (2017) [24] Palestine	To analyze the psychometric properties of the Psychiatric Nurses Methods of Coping Questionnaire (PNMCQ) - Arabic version	Reliability and validity study	99 PNs^2^	The PNMCQ demonstrated validity and reliability for measuring coping skills of Jordanian psychiatric nurses
Sargazi *et al*. (2018) [25] Iran	To assess the impact of a stress management program on the professional competences of psychiatric nurses	Quasi- experimental with control group and cluster randomization	70 PNs^2^	Higher levels of professional competence in the intervention group compared to control
Dickens *et al*. (2019) [26] Australia	To review mental health nurses’ knowledge, skills, experience, attitudes, or training needs related to routine physical healthcare. To assess the effectiveness of mental health nurses’ delivery of routine physical healthcare to mental health patients	Systematic review with 51 studies	7549 MHNs^1^	To improve training of nurses to provide physical healthcare to individuals with mental disorders. It is necessary to determine the appropriate content for such preparation and evaluate its effectiveness. It is also important to develop approaches congruent with the needs and preferences ofpatients
Hartley *et al*. (2019) [9] Canada	To explore continuing education interventions for nurses working in psychiatry	Systematic Review with 16 studies	>1100 nurses working in psychiatric	Educational initiatives help to ensure competent nursing practice. Evidence suggests that continuing education increases knowledge, confidence and skills or improves attitudes. However, more rigorous research into continuing education intervention is needed
Hasan and Abulattifah (2019) [27] Saudí Arabia	To examine psychiatric nurses’ knowledge, attitudes and practices regarding the use of physical restraint	Cross‐sectional design	110 PNs^2^	Participants had moderate knowledge, attitudes and practices: less than half recognized alternative approaches to restraint and most did not understand the reasons for restraint
Kurebayashi (2019) [28] Japan	To clarify the associations of occupational stress, self-focus, and other-consciousness with nursing skills, identify differences between general and psychiatric nurses	Cross-sectional study	317 PNs^2^	The other-consciousness and occupational stress predicted skills in both groups, while lower rumination and higher reflection scores only in mental health nurses
Kurebayashi (2021) [29] Japan	To clarify the associations between self‐focus, self‐ compassion, and nursing competency among psychiatric nurses	Cross-sectional study	433 PNs^2^	Lower fantastic aspect and higher internal aspect in other- consciousness, higher reflection in the rumination-reflection questionnaire, and lower negative subscales of the self-compassion scale predicted higher oriented problem-solving behavior in nursing practice score
Rivera Vicente (2021) [30] Spain	To develop a tool to assess the competences of the mental health nurses during their training program residency	Reliability and validity study	40 MHNs^1^	Validated the scale ECOEnFSM. to assess mental health nursing competences in Spain
Yıldırım Üşenmez and Gümüş (2021) [31] Turkey	To evaluate the effect of empathy skills on attitudes and practices towards the use of physical restraint	Cross-sectional study	100 nurses working in psychiatric	Moderate knowledge, attitudes and empathy skills, and attitudes towards physical restraints were appropriate and practices ideal. High correlation was observed between empathy scores and higher educational levels, as well as a weak correlation between empathy scores and attitude scores
Dickens *et al*. (2022) [32] United Kingdom	To measure the attitudes of UK mental health nurses. effectiveness of interventions and relationships between attitudes, other variables/constructs and practice	Systematic Review with 42 studies	>4.600 MHNs^1^	Negative attitudes were most common toward borderline personality disorder, substance misuse, and acute mental health presentations. Interventions improved attitudes but there were little evidence of sustainability and few studies of attitude- practice links
Ezeobele *et al*. (2022) [33] USA	To assess knowledge and attitudes of psychiatric nurses toward electroconvulsive therapy (ECT)	Cross‐sectional study	158 PNs^2^	The knowledge of ECT ranged from 45% to 99% and positive attitudes ranged from 21% to 96%, showing improvement compared to previous studies
Merrick *et al*. (2022) [34] Australia	To explore the perceptions and attitudes of mental health nurses towards the use of alcohol and other drugs by mental health users	Systematic review with 12 studies	>430 MHNs^1^ and nurses working in psychiatry	Attitudes towards dual diagnosis were often negative, likely impacting care quality and treatment outcomes. The lack of recent studies reveals a need for up-to-date knowledge
Sheikhbahaeddinzadeh *et al*. (2022) [35] Iran	To investigate and critique the psychometric properties of some psychiatric nursing clinical competency assessment instruments	Systematic review with 6 studies	Nurses working in psychiatry and postgraduate psychiatric nurses	Most tools did not adequately report psychometric properties. This is not designed as a tool for postgraduate psychiatric nursing. To assess the competency of psychiatric nursing postgraduate, a tool tailored to the cultural and social context and with acceptable psychometric properties is necessary
Song *et al*. (2022) [36] Korea	To introduce and evaluate the effectiveness of a competency’s education program for psychiatric nursing	Quasi- experimental design	64 PNs^2^	The competency-based educational program has demonstrated to enhance the overall professional nursing competence, professional self- growth, management and teaching competences of both groups
Han *et al*. (2023) [37] Taiwan	To explore possible association between personal and external factors with spiritual care competency in mental health nurses	Cross-sectional study	239 MHNs^1^	Personal and external factors may influence MHNs’ self-perception of spiritual care competency. Significant positive correlations between spiritual care competency and previous participation in spiritual care education programs, longer working experience, higher education level, and the personality traits of “Conscientiousness”, “Agreeableness”, “Extraversion”, and “Openness/Intellect”

^1^MHNs, Mental Health Nurses. ^2^PNs, Psychiatric Nurses.

The analysis included a sample of over 17,500 participants comprising Mental Health Nurses, Psychiatric Nurses, and Nurses working in psychiatry.

### Study Variables

The primary focus of the analysis was on assessment tools used to evaluate professional competences of Mental Health Nurses in training programs or clinical practice (n = 23 studies). Among systematic reviews (n = 6), four included studies using non-validated, ad-hoc instruments developed by researchers; two included only validated assessment tools. Of the remaining primary studies (n = 17), fourteen used validated assessment tools and three used instruments developed by researchers.

### Study Findings

Assessments covered both general competences in mental health nursing and specific competences related to functions or domains. The most frequently evaluated areas were: physical healthcare (n = 5), including diabetes, tobacco use, sexual health, and general physical condition; and general functions of mental health nursing (n = 5), including assessment, interviewing, diagnosis, intervention, evaluation, kindness, cooperation, communication, nurse-patient relationship, psychological support, case management, and research. Other commonly assessed topics included addictions, therapeutic restraint, psychiatric emergencies, medication, self-harm, and suicide.

All identified tools focused on measuring knowledge, skills, and attitudes. After eliminating duplicates, a total of 100 tools were examined. Among these, validated assessment tools (n = 66) outnumbered non-validated or researcher-developed assessment tools (n = 34). Validated tools were most commonly associated with specific competences such as therapeutic restraint or physical health. Examples of such tools include the Attitudes towards Containment Measures Questionnaire (ACMQ) and the Physical Health Attitude Scale for Mental Health Nurses (PHASe).

However, a significant number of tools also targeted general and crosscutting competences. Notable examples include the Clinical Competency of Mental Health Nursing (CCMHN), the Psychiatric Nursing Performance Appraisal Instrument (PsychNPAI), the Clinical Nursing Competence Questionnaire (CNCQ-22) and the Mental Health Nursing Competence Assessment Scale (ECOEnfSM) (Table 4, Ref. [9, 16, 17, 18, 19, 20, 21, 22, 23, 24, 25, 26, 27, 28, 29, 30, 31, 32, 33, 34, 35, 36, 37]).

**Table 4.** **Summary of training programs and assessment tools for competences of Mental Health Nurses**.

Author (year)	Topic	Competences	Assessment tools
Robson (2012) [16]	Physical healthcare	Attitudes	- Physical Health Attitude Scale for Mental Health Nurses
Hemingway (2013) [17]	Diabetic care	Knowledge	- Knowledge questionnaire (researcher developed)
Park (2014) [18]	Addiction	Skills and Attitudes (positive self-worth, comprehension, environment comprehension, motivation, perception of personal competency, communication skills, addiction comprehension and addiction management skills)	- Expert Competency Model Scale of Psychiatric Mental Health Nurses for Preventing Adolescent Addictive Behavior
White (2014) [19]	Physical healthcare	Knowledge and attitudes	- Knowledge questionnaire and an attitude Likert scale (researcher developed)
Hemingway (2015) [20]	Diabetic care	Knowledge and skills	- Knowledge questionnaire (researcher developed)
Matsuda (2015) [21]	Psychosis (treatment, symptoms, acceptance of illness, improve their medication adherence, stress)	Knowledge, skills and attitudes (psychoeducation, self-efficiency, attitudes, perception of psychoeducation and motivation)	- Work Motivation Measurement Scale for Nurses (Sano, 2005). - Evidence based Practice Attitude Scale Japanese version (Okumura, 2010). - General Self Efficacy Scale (Sakano, 1989). - Knowledge of Illness and Drugs Inventory (Maeda, 1992). - Nurses´ Perception of Psychoeducation Practice (researcher developed)
de Almeida Vieira Monteiro (2016) [22]	Multicultural	Knowledge, skills and attitudes (awareness)	- Multicultural Mental Health Awareness Scale version Portuguese: cultural competences (Khawaja, 2009)
Krawitz (2004) in Dickens (2016) [23]	Border line personality	Knowledge and attitudes (willingness, optimism, enthusiasm, confidence, theoretical knowledge and clinical skills)	- Survey questionnaire
Commons (2008) in Dickens (2016) [23]	Border line personality Self-harm	Attitudes (trust, empathy, intervention, referral)	- Attitudes Towards Deliberate Self Harm Questionnaire (McAllister, 2002)
Stringer (2015a) in Dickens (2016) [23]	Border line personality Self-harm Suicide	Skills and attitudes (therapeutic relationships, trust, empathy, intervention, referral)	- Scale to Assess Therapeutic Relationships in Community Mental Healthcare Clinician version (Guire Snieckus, 2007) - Suicidal Behavior Attitude Scale (Botega, 2005) - Attitudes Towards Deliberate Self Harm Questionnaire (McAllister, 2002)
AL-Sagarat (2017) [24]	Coping strategies	Skills	- Psychiatric Nurses Methods of Coping Questionnaire (McElfatrick, 2000)
Sargazi (2018) [25]	Anxiety and relaxation	Knowledge, skills and attitudes (clinical care, leadership, interpersonal relationships, ethical/legal practice, professional development, teaching, critical thinking, research attitude)	- Competency Inventory for Registered Nurse (Liu, 2009).
Brimblecombe (2005) in Dickens (2019) [26]	Physical healthcare	Knowledge	- Knowledge questionnaire (researcher developed)
Nash (2005) in Dickens (2019) [26]	Physical healthcare	Knowledge and skills	- Knowledge and skills questionnaire (researcher developed)
Artzi-Medvdik (2006) in Dickens (2019) [26]	Breastfeeding in women with schizophrenia diagnosis.	Knowledge and attitudes	- Knowledge and Attitudes to Breastfeeding (Freed, 1995)
Robson (2006–2007) Haddad (2016) Wynaden (2014) in Dickens (2019) [26]	Physical healthcare	Attitudes	- Physical Health Attitude Scale for Mental Health Nurses (Robson, 2012)
Delaney (2009) in Dickens (2019) [26]	Physical healthcare	Knowledge	- Knowledge questionnaire (researcher developed)
Dorsay (2009) in Dickens (2019) [26]	Sexual health	Knowledge	- Knowledge questionnaire (researcher developed)
Hughes (2009) in Dickens (2019) [26]	Sexual health (HIV/AIDS)	Knowledge	- Knowledge questionnaire (researcher developed)
Nash (2009) in Dickens (2019) [26]	Diabetes	Knowledge	- Knowledge questionnaire (researcher developed)
Sharp (2009) in Dickens (2019) [26]	Smoking	Skills	- Questions assessing intervention skills followed Ask, Advise, Assess, Assist, Arrange recommendations (Morris, 2009)
Howard (2010) in Dickens (2019) [26]	Physical healthcare	Knowledge and skills	- Knowledge and skills questionnaire (researcher developed)
Happell (2013-15) in Dickens (2019) [26]	Physical healthcare	Knowledge	- Knowledge questionnaire (researcher developed)
Happell (2013-2015) in Dickens (2019) [26]	Physical healthcare	Skills and attitudes	- Physical Health Attitude Scale for Mental Health Nurses (Robson, 2012). - Nurse with other staff on the physical health of user’s questionnaire (researcher developed)
Terry (2013) in Dickens (2019) [26]	Physical healthcare	Knowledge	- Knowledge questionnaire (researcher developed)
Osborn (2015) in Dickens (2019) [26]	Physical healthcare	Skills	- Physical Assessment Skills Inventory (Birks, 2012; Giddens, 2007) - Barriers to Registered Nurses’ Use of Physical Assessment Scale (Douglas, 2014)
Magor-Blatch (2016) in Dickens (2019) [26]	Smoking	Attitudes	- Attitudes toward Smoking Scale (Shore, 2000)
Sung (2016) in Dickens (2019) [26]	Sexual health	Knowledge, skills and self-efficacy	- Knowledge of sexual health questionnaire (researcher developed) - Attitude toward sexual health questionnaire (researcher developed) - Self-efficacy for sexual health questionnaire (researcher developed)
Bressington (2016–2017) in Dickens (2019) [26]	Physical healthcare	Attitudes	- Physical Health Attitude Scale for Mental Health Nurses (Robson, 2012)
Ganiah. (2017) in Dickens (2019) [26]	Physical healthcare	Attitudes	- Physical Health Attitude Scale for Mental Health Nurses (Robson, 2012)
Chee (2018) in Dickens (2019) [26]	Physical healthcare in First Episode Psychosis care	Attitudes	- Physical Health Attitude Scale for Mental Health Nurses (Robson, 2012)
Clancy (2018) in Dickens (2019) [26]	Physical healthcare	Attitudes	- Physical Health Attitude Scale for Mental Health Nurses (adapted Robson, 2012; Happell, 2013)
Parel (2018) in Dickens (2019) [26]	Smoking	Knowledge and attitudes	- Knowledge and attitudes questionnaire (researcher developed)
Quinn (2018) in Dickens (2019) [26]	Sexual health	Skills	- Skill questionnaire (researcher developed)
Sharma (2018) in Dickens (2019) [26]	Smoking	Skills	- Skill questionnaire (based on Ford, 2015)
Arkan (2008) in Dickens (2019) [26]	Electroconvulsive therapy	Knowledge and attitudes	- Observation and satisfaction form and survey (researcher developed)
Redhead (2011) in Hartley (2019) [9]	Care plans	Knowledge and attitudes	- Knowledge questionnaire (researcher developed)- Attitude to Psychosocial Intervention scale (Richards, 1999)
Tsai (2011) in Hartley (2019) [9]	Suicide	Knowledge	- Awareness of suicide warning signs (researcher developed)
Aminoroaia (2014) in Hartley (2019) [9]	Mental Health Nursing	Knowledge and attitudes	- Knowledge questionnaire (researcher developed) - Attitude questionnaire (researcher developed)
Hung (2015) in Hartley (2019) [9]	Critical thinking	Attitudes (analysis open-minded, curiosity, reflection)	- Critical Thinking Disposition Inventory (Yeh, 1998)
Attari (2017) in Hartley (2019) [9]	Medication and electroconvulsive therapy	Knowledge and attitudes	- Knowledge questionnaire (researcher developed) - Attitude questionnaire (researcher developed)
Ragaisis (2017) in Hartley (2019) [9]	Motivational interviewing	Skills	- Questionnaire (researcher developed)
Russell (2017) in Hartley (2019) [9]	Substance use	Knowledge	- Perceived competency questionnaire (researcher developed) (based on Lakeman, 2010)
Zuaboni (2017) in Hartley (2019) [9]	Recovery-oriented care (psychosis)	Knowledge (social inclusion, motivational interviewing, therapeutic relationships)	- Recovery Self Assessment scale (Zuaboni, 2015; O’Connell, 2005)
Hasan (2019) [27]	Physical restraints	Knowledge and attitudes	- Level of knowledge about restraint scale (Janelli, 1992) - Attitudes and practice of nurses towards the use of restraints
Kurebayashi (2019) [28]	Mental Health Nursing	Attitudes and skills (occupational stress, Other-consciousness, Self- focus, assessment, diagnosis, intervention, evaluation, psychological support)	- Rumination Reflection Questionnaire version Japanese (Takano, 2008) - Brief Job Stress Questionnaire (Shimomitu)- Self Evaluation Scale from the Oriented Problem Solving Behavior in Nursing Practice (Sadahiro, 2002) - Other consciousness Scale (Tsuji, 1993)
Kurebayashi (2021) [29]	Other-consciousness, Self- compassion, Self-focus and Nursing Competency	Attitudes and skills	- Rumination Reflection Questionnaire version Japanese (Takano, 2008) -Self Compassion Scale version Japanese (Arimitsu, 2014)-Self Evaluation Scale from the Oriented Problem- Solving Behavior inNursing Practice (Sadahiro, 2002) -Other Consciousness Scale (Tsuji, 1993)
Rivera Vicente (2021) [30]	Assessment, Nursing Diagnosis, Intervention, Evaluation, Research, Clinical Management, Teaching and Interpersonal Relationships	Knowledge, skills and attitudes	- Assessment competences of Mental Health Nurses in Specialized Health Training Programs (Rivera, 2021)
Yıldırım Üşenmez (2021) [31]	Physical restraints (therapeutic immobilization)	Knowledge, skills and attitudes	- Empathy skills scale (Dökmen, 1988) - Knowledge, attitudes, and practices of staff towards physical restraints questionnaire (Suen, 1999)
Anderson (2000) in Dickens (2022) [32]	Suicidal behavior	Attitudes	- Suicide Opinion Questionnaire (Domino, 1982)
Hannigan *et al*. (2000) in Dickens (2022) [32]	Negative attitudes to service users	Attitudes	- Maslach Burnout Inventory (Maslach, 1986)
Mistral (2002) in Dickens (2022) [32]	Psychiatric emergencies	Attitudes	- The Attitude Measure
Whittington (2002) in Dickens (2022) [32]	Aggression	Attitudes	- Perception of Aggression Scale (Jansen, 1997)
Markham (2003) in Dickens (2022) [32]	Borderline personality disorders	Attitudes	- Treatment Optimism scale (Dagnan, 1998) -Beliefs About Dangerousness (Link, 1987) -Modified Social Distance Scale (Ingamells, 1996)
Richmond (2003) in Dickens (2022) [32]	Substance abuse	Attitudes	- Substance Abuse Attitude Survey (Chappel, 1985)
Baker (2005) Munro (2009) in Dickens (2022) [32]	Psychiatric emergencies	Attitudes	- Attitudes Towards Acute Mental Health Scale
Patel (2005)Patel (2009) in Dickens (2022) [32]	Depot medication MHN prescribing of psychotropic medication	Attitudes	- Attitude Questionnaire (researcher developed)
Bowers (2006) Bowers (2008) Bowers (2015) James (2017) in Dickens (2022) [32]	Personality disorders	Attitudes	- Attitude to Personality Disorder Questionnaire
Bowers (2006) Bowers (2008) Whittington (2009) in Dickens (2022) [32]	Physical restraints (therapeutic immobilization)	Attitudes	- Attitudes towards Containment Measures Questionnaire
Bradshaw (2007) in Dickens (2022) [32]	Schizophrenia	Knowledge and Attitudes	- Knowledge and Attitude questionnaire (researcher developed)
Harris (2007) in Dickens (2022) [32]	Depot medication	Attitudes	- Staff Attitude to Neuroleptic Treatment Inventory
Munro (2007) in Dickens (2022) [32]	Co-morbid substance uses and mental health problems	Knowledge and Attitudes	- Co-Morbidity Problems Perceptions Questionnaire (adapted from Alcohol Problems Perceptions Questionnaire) (Cartwright, 1980)
Patterson (2007a,2007b) Hosie (2018) in Dickens (2022) [32]	Self-harm	Attitudes	- Self Harm Antipathy Scale
Wood (2007) in Dickens (2022) [32]	Electroconvulsive therapy	Knowledge and attitudes	- ECT attitude scale
Bowers (2010) in Dickens (2022) [32]	Attitudes to Locked Doors on acute mental health wards	Attitudes	- Attitudes towards Containment Measures Questionnaire
Guise (2010) Morris (2011) in Dickens (2022) [32]	Mental illness	Attitudes	- Community Attitudes towards the Mentally Ill (Taylor, 1981)
Davies (2014) Ebrahim (2016) Lamph (2017)Acford (2019) in Dickens (2022) [32]	Personality disorders	Knowledge, attitudes and skills	- Personality Disorders Knowledge, Attitudes and Skills Questionnaire (Bolton, 2010)
Pettit (2016) in Dickens (2022) [32]	Physical restraints (therapeutic immobilization)	Attitudes	- Attitudes towards Containment Measures Questionnaire (Bowers, 2004)
Lavelle (2017) in Dickens (2022) [32]	Depot medication Psychiatric emergencies	Knowledge and Attitudes	- Knowledge and Attitude questionnaire (researcher developed)
Dickens (2018) in Dickens (2022) [32]	Borderline personality disorders	Attitudes	- Borderline Personality Disorder Cognitive Attitudes Inventory (Bodner, 2015) - Emotional Attitudes Inventory (Bodner, 2015)
Hosie (2018) in Dickens (2022) [32]	Self-harm	Attitudes	- Attitudes to Self-cutting Management Scale - Attitudes towards Containment Measures Questionnaire (Bowers, 2004)
Georgieva (2019) in Dickens (2022) [32]	Mental Health Legislation	Attitudes	- Mental Health Legislation Attitudes Scale (researcher developed)
Laker (2019) in Dickens (2022) [32]	Negative attitudes to users	Attitudes	- Maslach Burnout Inventory (Maslach, 1986)
Rogers (2019) in Dickens (2022) [32]	Spirituality	Attitudes	- Modified spirituality in education attitudes questionnaire (Prentis, 2014)
Sandford (2020) in Dickens (2022) [32]	Suicide	Attitudes	- Attitudes to Suicide Prevention Scale (Herron, 2001)
Ezeobele (2022) [33]	Electroconvulsive therapy	Knowledge and attitudes	- The Questionnaire on Attitudes and Knowledge
Siegfried (1999) in Merrick (2022) [34]	Addiction Dual pathology	Attitudes	- Attitudes questionnaire (researcher developed)
Molina-Mula (2018) in Merrick (2022) [34]	Addiction Dual pathology	Attitudes	- Seaman-Mannello Scale (Seaman, 1978)
Bondy (1997) in Sheikhbahaeddinzadeh (2022) [35]	Mental health nursing	Knowledge, skills and attitudes (basic knowledge, critical thinking, nursing process, nursing interventions, communication skills, professional socialization behaviors, self-evaluation)	- Psychiatric Nursing Performance Appraisal Instrument
Mohtashami (2014) in Sheikhbahaeddinzadeh (2022) [35]	Mental health nursing	General and specific competences	- Clinical competency in mental health nursing students
Moskoei (2017) in Sheikhbahaeddinzadeh (2022) [35]	Mental health nursing	Skills	- Clinical Competency of Mental Health Nursing (CCMHN)
Chen (2018) in Sheikhbahaeddinzadeh (2022) [35]	Mental health nursing	Knowledge and skills (use of resources, social and economic psychological support, rehabilitation activity)	- Case Management Competence Scale
Feng (2018) in Sheikhbahaeddinzadeh (2022) [35]	Mental health nursing	Attitudes (sense of responsibility, kindness, cooperation capacity)	- Mental Health Objective Structured Clinical Examination
Stockman (2019) in Sheikhbahaeddinzadeh (2022) [35]	Mental health nursing	Attitudes and skills (mental state, therapeutic communication and nursing relationship)	-Questionnaire to assess nursing competences for the care of people with psychiatric disabilities in a hospital environment
Song (2022) [36]	Mental health nursing	Knowledge and skills (psychiatric emergency, psychopharmacology, law and ethics, diagnosis, interview skills and physical healthcare.)	- Clinical Nursing Competence Questionnaire (Lee-Hsieh, 2003)
Han (2023) [37]	Spirituality care	Knowledge and attitudes (educational programs, awareness, kindness, extroversion, emotional stability, openness/intelligence, spiritual care, psychological support, referral, and communication)	- Big five Mini Markers questionnaire (Thompson, 2008) - Spiritual care competency scale (Leeuwen, 2009)

Across the twenty-three studies reviewed, evidence consistently demonstrated that educational interventions and assessment tools play a critical role in enhancing the knowledge, skills and attitudes of mental health nurses in key areas of practice. Workshops and training programs were particularly effective in delivering immediate knowledge gains (such as in diabetes care, electroconvulsive therapy, and personality disorders) while also fostering more positive attitudes toward physical healthcare and complex clinical presentations.

Several studies further contributed by developing and validating competency assessment instruments with robust psychometric properties. These tools proved valuable for evaluating multicultural competence, addiction-related skills, and broader mental health nursing competences.

Despite these advances, the literature underscores the need for more rigorous and culturally adapted tools, as well as longer, tailored training programs that address the specific needs of both patients and professionals. Such improvements are essential to enhance clinical competence and quality of care, given that short-term knowledge gains do not always lead to long-term changes in practice or clinical skills.

## Discussion

Training programs evaluated using these tools have demonstrated their effectiveness in fostering competence acquisition among Mental Health Nurses. However, identifying programs specifically designed for practicing nurses, rather than nursing students, remains difficult. The existing programs vary significantly across regions, employing diverse approaches that are typically short in duration (ranging from days to months), theory-oriented, and prioritize knowledge acquisition over practical skill development. Similarly, many assessment tools lack uniformity, often being applicable across multiple health professions and failing to provide comprehensive coverage [9, 10, 21, 38].

As a result, there is critical need to unify and standardize training programs and competence evaluation methods. While achieving global consistency may be ambitious due to cultural and contextual differences, establishing national-level standardization and objectivity is both feasible and necessary.

The studies primarily concentrated on populations with psychosis, borderline personality disorder, and severe mental illness, often overlooking other significant areas such as addictions or eating disorders. Moreover, it was difficult to identify research conducted exclusively with specialist mental health nurses. Even among the included studies, it was frequently unclear whether participants were formally trained specialists or nurses with general psychiatric experience. This ambiguity hinders meaningful comparisons of roles and competences across different countries and training systems.

Most of the reviewed studies assessed specific competences related to physical health problems (e.g., diabetes, tobacco use, exercise, sexual health) or the management of critical situations (e.g., suicide risk, self-harm, therapeutic restraints), which are considered essential in psychiatric nursing. In contrast, only a minority addressed transversal competences, such as therapeutic communication, critical thinking, or researching and teaching skills. This imbalance is likely due to the simplicity and lower cost of cross-sectional designs, which predominantly focus on knowledge and attitudes–areas which are easier and less resource-intensive to evaluate compared to skills. Skills assessment often requires simulations, role playing, or 360° evaluations, which are more complex and costly. Consequently, the most frequently assessed competences were those directly linked to clinical care, with limited attention given to teaching, managerial, or research roles.

The overemphasis on knowledge and attitude assessments, without adequate evaluation of practical skills, risks producing clinicians who are theoretically knowledgeable but lack essential hands-on competence. This discrepancy can compromise patient safety and care quality, leading to ineffective clinical decision- making, diminished procedural proficiency, and lower patient satisfaction or adherence to treatment plans. Therefore, it is imperative to implement a balanced assessment framework which integrates knowledge, attitudes and skills and thereby ensuring comprehensive clinical preparedness.

The findings reveal a lack of standardized, culturally adapted, and psychometrically robust tools tailored to postgraduate Mental Health Nursing. Most existing instruments are designed for general or undergraduate populations, underscoring the need for harmonization of training programs and competence evaluation at both national and international levels.

Equally important is the cultural adaptation of tools and interventions. This includes addressing language barriers, cultural norms in healthcare practices, and variations in educational backgrounds. The cross-cultural validity of tools often depends on their translation and adaptation. For example, Sheikhbahaeddinzadeh *et al*. [35] highlight studies adapted to the Iranian cultural context. However, this review found no studies explicitly addressing cultural competency, a crucial aspect of delivering effective and culturally sensitive care. The assessment tool developed by Rivera Vicente, while more aligned with the real training and clinical context of Mental Health Nurses in Spain, has not been translated into English, limiting its applicability outside Spanish- speaking contexts. These challenges underscore the complexity of adapting assessment instruments and the necessity for culturally sensitive modification to ensure successful integration into routine practice [30, 35, 39].

This review identified several tools for assessing nursing competences in mental health, including both validated and non-validated instruments, ranging from those targeting single competences to those covering longitudinal nursing functions. Due to the inclusion of systematic reviews, the 23 studies analyzed encompassed a broader set of 103 studies. Among these, validated assessment tools (n = 77) outnumbered non-validated tools (n = 26). After eliminating duplicates, 100 assessments tools were identified, with validated tools (n = 66) exceeding not-validated or research developed tools (n = 34). The presence of a significant number of non-validated tools (34%) limits the generalizability of their findings. Many of these were author-developed knowledge questionnaires, likely due to tehri simplicity and resemblance to theoretical examinations, whereas tools measuring skills or attitudes are more complex and less frequently used. Non-validated scales were often employed in methodologically simple and low-cost studies.

Conversely, 74.7% of the studies used validated scales, and 66% of the total instruments detected were validated, enabling greater reproducibility, data extrapolation, and methodological rigor. The most frequently used scales were the Physical Health Attitude Scale for Mental Health Nurses (PHASe, 8.7%, n = 9) and the Attitudes towards Containment Measures Questionnaire (ACMQ, 5.8%, n = 6). These scales reflect traditional psychiatric nursing functions, such has physical health and restraint, reinforcing their importance but also perpetuating a classical role that may overshadow the involvement of nurses in other essential domains.

The tools varied in length and dimensionality, ranging from 17 to over 70 items distributed across 6–8 dimensions. This disparity highlights the heterogeneity in competence assessments. Even when reliability data were reported, the results were often inconsistent or incomplete for establishing validity [35]. Longer tools tended to assess both specific (clinical) and transversal (educational, managerial, research) competences, whereas shorter tools focused narrowly on specific themes.

Overall, the evidence demonstrates robust methodological quality, with a high proportion of systematic reviews adhering to international standards (such as PRISMA-ScR and COSMIN) [23, 26, 32, 34], and psychometric validation studies employing rigorous statistical analyses, including factor analysis and reliability testing [22, 24, 35]. The primary studies, predominantly cross-sectional and quasi-experimental, were distinguished by their use of validated instruments and representative samples of mental health nursing professionals across diverse international contexts. However, recurrent limitations were noted, particularly in the quality of some ad-hoc developed tools and educational intervention designs, such as the absence of control groups in some cases and limited short-term follow-up. These limitations suggest caution when generalizing the sustainability of behavioral changes [21, 25, 36].

This scoping review is novel, particularly in Spain, where similar reviews are scarce. It provides a foundation for future research aimed at developing homogeneous, culturally adapted, psychometrically robust tools for postgraduate mental health nursing.

Future studies could include longitudinal descriptive analyses to compare differences in training or competence acquisition across universities, hospitals, regions, or countries, or to determine whether competence levels influence other care quality indicators, such as patient satisfaction, the use of mechanical restraints, or admission rates. Quasi-experimental studies could also evaluate the effectiveness of Specialized Health Training Programs for Mental Health Nurses. Another possible and currently expanding line of research is the assessment of acquired competences through high- fidelity simulation and compared with clinical practice.

## Conclusions

All tools included in this review offer valuable insights into competence assessment. However, there remains a pressing need for instruments specifically tailored to postgraduate psychiatric/mental health nursing and residency contexts. It is recommended that multiple scales and complementary methods, such as simulations and feedback evaluations, be integrated into the competence assessment of Mental Health Nurses.

## Disclosures

This paper does not contain copyright materials.

## Availability of Data and Materials

Not applicable.
